# Burden, Regional Trends and Risk Factors of Breast, Cervical, Uterine, and Ovarian Cancers in Sub-Saharan Africa, 1990–2023: The Global Burden of Disease 2023

**DOI:** 10.3390/ijerph23040419

**Published:** 2026-03-26

**Authors:** Obasanjo Bolarinwa, Sharmake Gaiye Bashir, Joshua Okyere, Yusuf Hared Abdi, Hiba Abdi Salad, Olusegun Dada, Abdulwasiu Ojo Yusuff

**Affiliations:** 1 Department of Global Healthcare Management, York St John University, London EC1Y 8TZ, UK; 2 Demography and Population Studies Programme, University of the Witwatersrand, Johannesburg 2050, South Africa; 3 Faculty of Health Sciences and Tropical Medicine, Somali National University, 252 Mogadishu, Somalia; sharmake@st.snu.edu.so (S.G.B.); yusuf.hared@st.snu.edu.so (Y.H.A.); hibaasalad@gmail.com (H.A.S.); 4 School of Human and Health Sciences, University of Huddersfield, Queensgate, Huddersfield HD1 3DH, UK; joshuaokyere54@gmail.com; 5 Department of Computer Science, University of Lagos, Lagos 101017, Nigeria; olusegunedada1@gmail.com; 6 Department of Population Health and Policy, School of Health and Medical Sciences, City St George’s University of London, Cranmer Terrace, London SW17 0RE, UK; akoredey@yahoo.com

**Keywords:** cancer epidemiology, breast cancer, cervical cancer, sub-Saharan Africa, global burden of disease, temporal trends

## Abstract

**Highlights:**

**Public health relevance—How does this work relate to a public health issue?**

- Cancers are an increasingly important driver of morbidity and premature mortality among women in sub-Saharan Africa, showing a widening non-communicable disease burden during epidemiological transition.
- This study maps long-term trends and regional heterogeneity in breast, cervical, uterine and ovarian cancer incidence, mortality, and disability-adjusted life years across 48 countries from 1990 to 2023 using Global Burden of Disease estimates.

**Public health significance—Why is this work of significance to public health?**

- The findings show substantial and uneven growth in cancer burden across sub-Saharan Africa, with breast cancer rising sharply in absolute burden and cervical cancer remaining a dominant cause of cancer mortality in Eastern and Southern Africa.
- High mortality-to-incidence patterns and persistent regional inequities indicate gaps in prevention, early detection and treatment capacity, underscoring the need for targeted and region-specific cancer control responses.

**Public health implications—What are the key implications or messages for practitioners, policy makers and/or researchers in public health?**

- Expanding equitable access to HPV vaccination, organised screening and timely diagnostic and treatment services is essential, with prioritisation tailored to regional burden profiles and age-specific risk patterns.
- Prevention strategies should address dominant modifiable risks, including unsafe sex for cervical cancer and metabolic and lifestyle risks for breast, uterine and ovarian cancers, alongside strengthened cancer surveillance and subnational monitoring to guide resource allocation.

**Abstract:**

**Background**: Sub-Saharan Africa is undergoing a rapid epidemiological transition marked by a growing burden of non-communicable diseases, including breast, cervical, ovarian, and uterine cancers, which constitute major causes of morbidity and mortality among women in the region; however, comprehensive assessments of long-term trends and regional heterogeneity remain limited. This study examines the burden and temporal trends of breast, cervical, ovarian, and uterine cancers across sub-Saharan Africa from 1990 to 2023. **Methods**: A retrospective ecological analysis was conducted using data from the latest Global Burden of Disease 2023 study. Age-standardised incidence rates, mortality rates, and disability-adjusted life year rates were estimated for breast, cervical, ovarian, and uterine cancers across 48 sub-Saharan African countries and four sub-regions. Temporal trends were assessed from 1990 to 2023, with percentage changes calculated to characterise epidemiological transitions. Geographic variation and age-specific patterns were examined to identify high-burden settings and priority populations. **Results**: Between 1990 and 2023, the burden of all four cancers increased substantially across sub-Saharan Africa, with significant regional and country-level heterogeneity. Breast cancer exhibited the largest absolute burden, with incidence increasing by over 120 percent and mortality by more than 80 percent, particularly in Central and Western Africa. Cervical cancer remained the leading cause of cancer-related mortality among women in Eastern and Southern Africa, despite evidence of stabilisation or decline in selected countries. Ovarian and uterine cancers demonstrated sustained upward trends, especially in Central Africa, with high mortality-to-incidence ratios indicating late diagnosis and limited treatment access. Across all cancer types, Central and Eastern sub-Saharan Africa consistently experienced the highest disability-adjusted life year burdens. **Conclusions**: The burden of the selected cancers in sub-Saharan Africa has increased markedly over the past three decades, with persistent regional inequities reflecting gaps in prevention, early detection, and treatment capacity. Strengthening cancer surveillance systems, expanding equitable access to screening and vaccination programmes, and improving diagnostic and treatment infrastructure are critical to reversing current trends. These findings provide region-specific evidence to guide cancer control priorities and resource allocation across sub-Saharan Africa.

## 1. Background

Sub-Saharan Africa (SSA) is currently undergoing a significant epidemiological transition, characterised by a “double burden” of disease [1,2,3]. Historically, cancer control in SSA has been deprioritised due to the overwhelming urgency of combating equally important infectious and parasitic diseases, including Human Immunodeficiency Virus/Acquired Immunodeficiency Syndrome (HIV/AIDS), malaria, and tuberculosis [4]. Notwithstanding the status quo, demographic shifts, urbanisation, sedentary lifestyle and consumption of alcohol and ultra-processed food are driving a surge in cancer incidence so much that, the health systems in SSA are ill-equipped to manage [5,6]. Among these, breast, cervical, uterine, and ovarian cancers represent a critical threat to the quality of life of women in the sub-region.

Evidence from the 2022 Global Cancer Observatory report shows that gynaecological cancers are among the top five cancers affecting women globally [7]. Specifically, breast cancer and cervix uteri cancer constituted 23.8% and 6.9% of all cancers among women, respectively [7]. In Africa, breast, cervix, uterus and ovarian cancers feature as part of the top five cancers among women [7,8]. Compared to the global picture, where breast cancer affects 23.8% of women, in Africa, it is higher (29.2%) [7]. The existing literature also indicates that in 2022, the age-standardised mortality rate for the selected cancers was highest in Eastern Africa (ASMR of 35.3 per 100,000, compared to 8.1 per 100,000 in Australia/New Zealand) [9].

Recognising the immense public health concern that these cancers pose [10,11], many SSA countries have instituted policies, frameworks and guidelines to usher their countries into a dawn of reduced cancer incidence and mortality. For example, Ghana has the national strategy for cancer control (NSCC) [12,13,14]; the country has also implemented a nationwide Human Papillomavirus (HPV) vaccination campaign as a strategy to eliminate cervical cancer [15]. Similarly, Rwanda implemented a national HPV vaccination programme to combat cervical cancer [16]. Tanzania and Malawi have also established *“national cancer registries, National Cancer Control Plans, early detection and screening policies and existing cancer treatment guidelines”* [17].

In the face of the existing cancer control and early detection/screening programmes, there are still disparities in the incidence, prevalence and mortality of cancers across SSA countries. It is important to know that this is not homogeneous across SSA. Being able to track changes across SSA countries over time is imperative for guiding future public health interventions, prioritizations, and the allocation of limited resources. Previous studies examining the burden of cancers have focused on the global burden without investigating the differences between countries in the SSA region. This situation obscures the distinct epidemiological patterns evolving within the continent’s sub-regions. To address this gap, the study utilises data from the 2023 Global Burden of Disease (GBD) study to provide a retrospective ecological analysis of breast, cervical, uterine, and ovarian cancer burden from 1990 to 2023. Examining trends in incidence, mortality, and disability-adjusted life years (DALYs) across 48 countries, this analysis aims to elucidate the shifting risk landscapes and identify high-priority zones for intervention.

## 2. Methodology

### 2.1. Data Source and Study Design

This retrospective ecological study assessed the breast, cervical, uterine, and ovarian cancer burden in SSA from 1990 to 2023 using data from the GBD 2023 study. The GBD database provides comprehensive, standardised estimates for disease incidence, mortality, and DALYs across 204 countries and territories, incorporating vital registration systems, cancer registries, epidemiological surveys, and statistical modelling to generate comparable health metrics [18,19].

### 2.2. Geographic Coverage and Population

The study encompassed all 48 sub-Saharan African countries, grouped into four sub-regions: Eastern SSA (14 countries), Western SSA (16 countries), Central SSA (9 countries), and Southern SSA (6 countries). Analysis included female populations across all age groups, with age-standardised rates calculated using the GBD reference population to enable valid temporal and cross-regional comparisons.

### 2.3. Health Outcomes and Metrics

Three primary health outcome indicators were extracted and assessed: age-standardised incidence rates (ASIRs) per 100,000 population, age-standardised mortality rates (ASMRs) per 100,000 population, and age-standardised DALY rates per 100,000 population. DALYs were computed as the sum of years of life lost (YLL) due to premature mortality and years lived with disability (YLD), quantifying both fatal and non-fatal disease burden. All estimates included 95% uncertainty intervals (UIs) to reflect statistical uncertainty resulting from data limitations and modelling assumptions.

### 2.4. Cancer Types Examined

Four major cancer malignancies were analysed: breast, cervical, ovarian, and uterine (endometrial cancer). These cancers were selected based on their substantial contribution to female cancer burden globally and their established risk factor profiles amenable to intervention.

### 2.5. Temporal Trend Analysis

Temporal changes in the ASIR, ASMR, and age-standardised DALY rates between 1990 and 2023 were quantified using relative percentage change. Percentage change was calculated as the proportional difference between the 2023 and 1990 estimates, divided by the 1990 baseline value, using the following formula:

(1) $$\mathrm{Percentage}\;\mathrm{change}\;(\%)=\left(\frac{{\mathrm{Rate}}_{2023}-{\mathrm{Rate}}_{1990}}{{\mathrm{Rate}}_{1990}}\right)\times 100$$

This approach was applied consistently across all cancer types, regions, and countries to characterise long-term epidemiological transitions over the 33-year study period. Temporal trends were further visualised using line graphs of annual age-standardised rates to identify patterns of increase, stabilisation, or decline across sub-regions.

### 2.6. Geographic and Age-Stratified Visualisation

Choropleth maps were generated to display spatial heterogeneity in the 2023 ASIR, ASMR, and DALY rates across sub-Saharan African countries for each cancer type. Age-specific incidence, mortality, and DALY distributions were plotted in five-year age bands to characterise age-related disease patterns and inform targeted screening strategies.

### 2.7. Risk Factor Attribution Analysis

Age-standardised DALY rates attributable to specific modifiable risk factors were extracted from the GBD comparative risk assessment framework. The risk factors analysed included high body mass index, high fasting plasma glucose, tobacco use, alcohol consumption, dietary risks, unsafe sex (for cervical cancer), and occupational exposures (for ovarian cancer). Attribution was conducted using population-attributable fractions derived from relative risk estimates and exposure prevalence data systematically compiled in the GBD methodology. Regional DALY burden attributable to each risk factor was calculated and stratified by cancer type to identify prevention priorities.

### 2.8. Data Analysis and Visualisation

All data extraction, processing, and statistical analyses were performed using R version 4.5.1 [20]. Data management, temporal trend calculations, and geographic stratification were conducted using standard R functions and libraries for epidemiological data analysis. Visualisation of geographic patterns, temporal trends, and risk factor attribution utilised R-based plotting packages to generate publication-quality figures.

### 2.9. Statistical Considerations

All estimates incorporated GBD standardised methodological approaches, including Bayesian meta-regression (DisMod-MR 2.1) for epidemiological parameter estimation, spatiotemporal Gaussian process regression for data-sparse locations, and cause-of-death ensemble modelling (CODEm) for mortality estimation. Uncertainty propagation ensured that 95% UIs captured parameter, sampling, and model specification uncertainty across all estimates.

## 3. Results

**Breast cancer incidence rates, death rates, and disability-adjusted life years in sub-Saharan Africa by region and country, 1990 to 2023**

Breast cancer burden in SSA has increased substantially over three decades, with regional incidence rates in 2023 ranging from 39.59 per 100,000 population in Southern Africa to 64.85 per 100,000 in Central Africa, representing a 122% increase in incidence since 1990. Mortality rates similarly escalated by 83%, reaching 29.68 per 100,000 across the region and 35.56 per 100,000 in Central Africa, while disease burden measured in DALYs increased by 86% to 1055.52 per 100,000 population. Country-level analysis reveals marked heterogeneity, with Equatorial Guinea (incidence: 107.01 per 100,000; mortality: 51.26 per 100,000) experiencing the highest burden, followed by Gabon and Côte d’Ivoire. In contrast, East African countries such as Somalia and Mozambique report substantially lower incidence rates. Temporal trends demonstrate disproportionate increases in Central Africa (186% incidence change; 135% mortality change), showing an epidemiological transition in this sub-region. In contrast, Southern Africa exhibits more modest increases (84% and 56%, respectively), as shown in Table 1.

**Figure 1:**
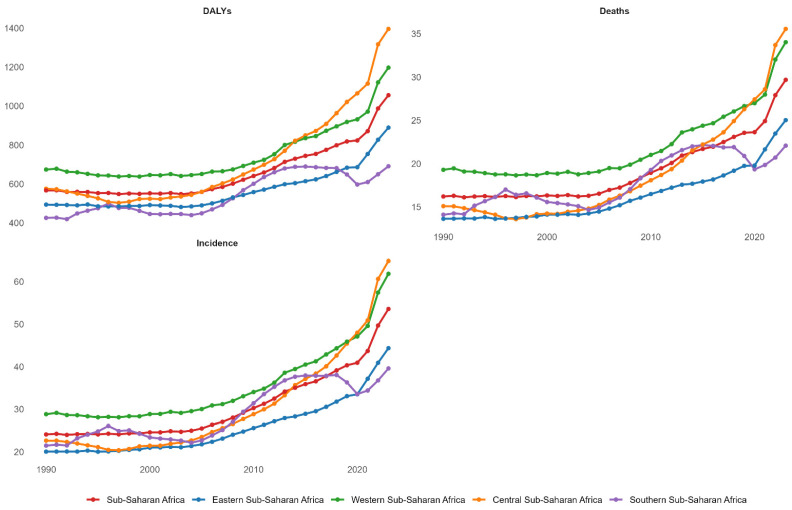
Temporal trends in age-standardised breast cancer incidence, mortality, and disability burden by sub-Saharan African region, 1990–2023.

Figure 1 shows that between 1990 and 2023, all SSA regions demonstrated marked upward trajectories across incidence, mortality, and disease burden metrics, with Central Africa (orange line) exhibiting the steepest acceleration, reaching 1400 DALYs per 100,000 and 35 deaths per 100,000 by 2023, while Western Africa (green line) followed closely with comparable trends. Eastern Africa (blue line) demonstrated the most modest growth, maintaining the lowest burden through 2023, despite experiencing exponential acceleration after 2010. In contrast, Southern Africa (purple line) showed intermediate burden levels with relative stabilisation in recent years. The incidence panel reveals a consistent divergence among regions, with the aggregate rates of Central, Western, and SSA converging toward 50–65 per 100,000 by 2023, contrasting sharply with the 20–45 per 100,000 range observed in 1990.

**Cervical cancer incidence rates, death rates, and disability-adjusted life years in sub-Saharan Africa by region and country, 1990 to 2023**

Table 2 shows that cervical cancer burden in SSA demonstrates substantial regional heterogeneity, with Eastern Africa experiencing the highest incidence (78.65 per 100,000) and mortality (37.03 per 100,000) rates in 2023, while Western Africa recorded the lowest burden (35.68 and 17.82 per 100,000, respectively), this shows more than two-fold variation across sub-regions. Country-level analysis reveals extreme disparities, with Eswatini exhibiting the highest incidence globally at 157.63 per 100,000 and catastrophic mortality at 69.44 per 100,000, generating 2999.15 DALYs per 100,000 population, while Malawi (135.87 incidence, 60.03 deaths), Zambia (112.59, 55.85), and South Sudan (99.27, 47.05) similarly demonstrate hyper-endemic burden. Temporal trends from 1990 to 2023 show divergent trajectories: Southern Africa experienced dramatic escalation with 120.5% incidence increase and 97.1% mortality increase, contrasting sharply with modest regional increases (30.3% and 15.0%, respectively). Declines were noticed in several countries including Mozambique (−34.2% incidence, −26.3% deaths), Tanzania (−18.3%, −22.1%), Madagascar (−2.3%, −6.6%), and Comoros (−20.6%, −24.0%). We identified persistently high case fatality ratios, particularly in Eswatini (44.0%), Malawi (44.2%), and South Sudan (47.4%).

**Figure 2:**
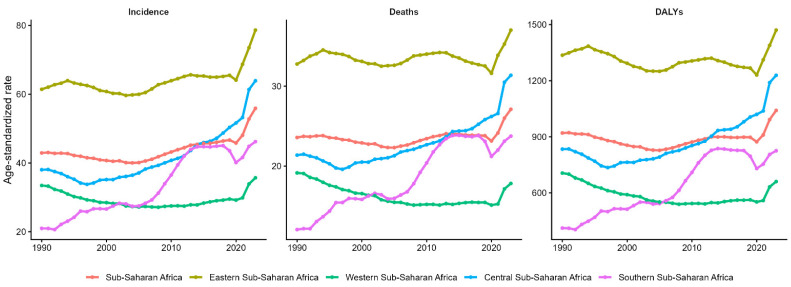
Temporal trends in age-standardised cervical cancer incidence, mortality, and disability burden by sub-Saharan African region, 1990–2023.

Cervical cancer burden in SSA from 1990 to 2023 shows distinct regional patterns: Eastern Africa maintains the highest incidence (60–80 per 100,000) and mortality (30–35 per 100,000) throughout the period, while Southern Africa experiences a dramatic post-2015 acceleration from 30 to 45 per 100,000 incidences and 15 to 25 per 100,000 mortalities. Western Africa and the regional aggregate remain relatively stable at 20–40 incidents per 100,000, whereas Central Africa shows a consistent upward trend, increasing from 35 to 70 incidents per 100,000 (Figure 2).

**Ovarian cancer incidence rates, death rates, and disability-adjusted life years in sub-Saharan Africa by region and country, 1990 to 2023**

As shown in Table 3, ovarian cancer burden in SSA increased by 109% for incidence and 97% for mortality from 1990 to 2023, with Eastern Africa showing the highest rates (10.52 per 100,000 incidences, 6.80 deaths) despite modest regional increases of 30%. Central Africa exhibits the steepest trajectory, with 148% incidence growth and 130% mortality increase, driven by Ethiopia and Equatorial Guinea (both >15 per 100,000 incidences with 300% increases). Southern and Western Africa demonstrate a lower but substantive burden (6.37 and 7.73 per 100,000, respectively), with incidence increases of 77% and 102%. Notable outliers include the Republic of Cabo Verde, with an extreme 462% incidence increase, the Republic of the Gambia (170% increase), and Senegal (176% increase), which likely reflects improved detection infrastructure and increased testing capacity. Mozambique remains the only country with a declining incidence (−9.1%), while most regions exhibit high case fatality ratios, particularly in Central African countries, where mortality-to-incidence ratios exceed 55–60%.

**Figure 3:**
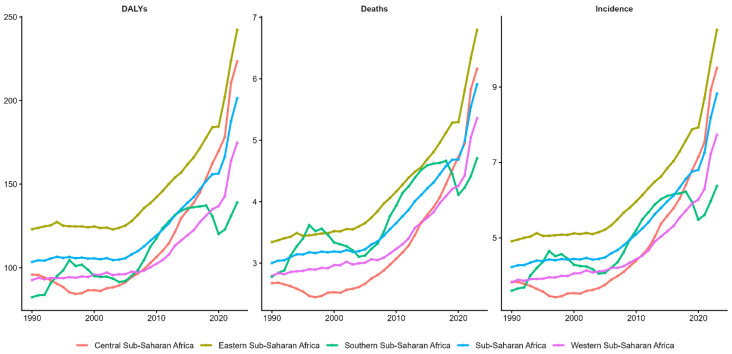
Temporal trends in age-standardised ovarian cancer incidence, mortality, and disability burden by sub-Saharan African region, 1990–2023.

The ovarian cancer burden from 1990 to 2023 shows a consistent escalation across all SSA regions, with Eastern Africa (yellow) maintaining the highest incidence (5–9.5 per 100,000) and mortality (3–6.5 per 100,000), resulting in 120–240 DALYs per 100,000, with accelerating post-2010 trends. Central Africa (red) and SSA aggregate (cyan) demonstrate parallel upward trajectories, rising from approximately 85–110 DALYs in 1990 to 180–200 DALYs by 2023, indicating a sustained increase in disease burden. Southern Africa (pink) exhibits a moderate burden (4–5.5 per 100,000 incidence) with 70–175 DALYs, while Western Africa (purple) remains at lower levels (3–4.8 per 100,000 incidences, 80–180 DALYs) with relatively gradual increases (Figure 3).

**Uterine cancer incidence rates, death rates, and disability-adjusted life years in sub-Saharan Africa by region and country, 1990 to 2023**

As shown in Table 4, uterine cancer burden in SSA increased by 72% for incidence and 38% for mortality from 1990 to 2023, with Eastern Africa demonstrating the highest rates (5.77 per 100,000 incidences, 2.51 deaths) and 81% and 43% increases, respectively. Central Africa exhibits the steepest growth trajectory, with a 115% incidence increase and a 69% mortality increase, particularly driven by the DR Congo (133% increase), Equatorial Guinea (232% increase), and São Tomé and Príncipe (90% increase). Western Africa exhibits a moderate burden (5.35 per 100,000 incidence) with a 61% increase, while Southern Africa shows more modest growth, with a 66% incidence increase and a 45% mortality increase, despite Botswana and South Africa showing substantial 75% and 77% incidence rises, respectively. Notable findings include Mauritania (9.04 incidents per 100,000, with a 150% increase), Ghana (9.22 incidents per 100,000), and Cabo Verde (7.82 incidents per 100,000), which contrasts with remarkably low rates in Mozambique and the Comoros (<2.5 incidents per 100,000).

**Figure 4:**
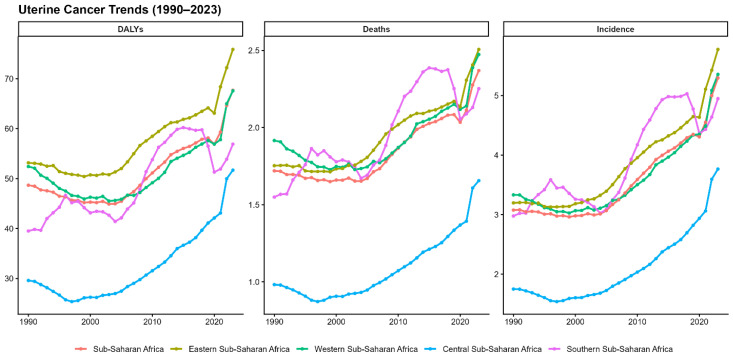
Temporal trends in age-standardised uterine cancer incidence, mortality, and disability burden by sub-Saharan African region, 1990–2023.

As shown in Figure 4, uterine cancer incidence from 1990 to 2023 demonstrates a modest overall escalation (72% incidence and 38% mortality increase) with striking regional divergence patterns. Eastern Africa (yellow) maintains the highest burden, with incidence rising from 5 to 7.5 per 100,000, mortality increasing from 1.8 to 2.8 per 100,000, and DALYs rising from 52 to 75, indicating steady linear growth throughout the period. Central Africa (cyan) exhibits the most dramatic trajectory, remaining negligible until 2000 before accelerating sharply after 2010, reaching 6.5 per 100,000 incidents and 2.5 per 100,000 mortalities, with 75 DALYs by 2023. This pattern suggests delayed disease emergence, improved detection infrastructure, or population ageing effects. Western Africa (green) and sub-Saharan Africa aggregate (red) display a relatively stable burden, ranging from 3 to 5.5 per 100,000 incidences, with modest increases. In contrast, Southern Africa (pink) exhibits volatility, with an initial decline from 1990 to 2000, followed by a resurgence after 2010, reaching 5 per 100,000 incidences and 2.2 per 100,000 mortalities.

**Spatial patterns of incidence, mortality, and disability-adjusted life years for breast, cervical, uterine, and ovarian cancers in sub-Saharan Africa (2023)**

**Figure 5:**
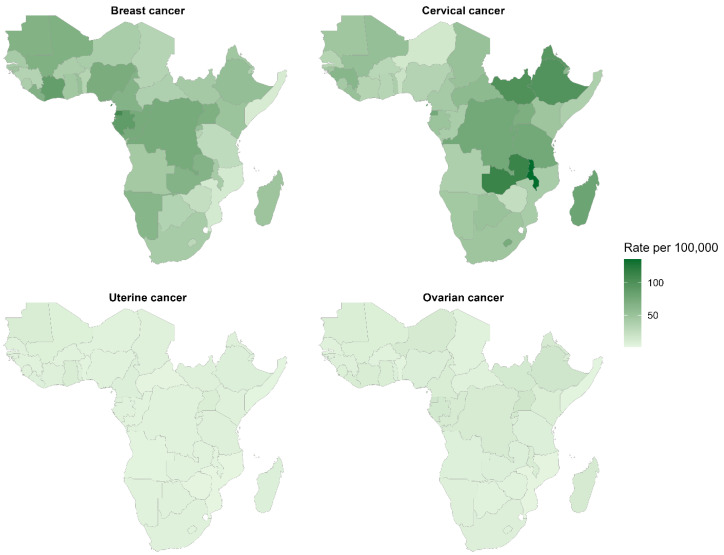
Geographic distribution of age-standardised incidence rates for breast, cervical, uterine, and ovarian cancers in sub-Saharan Africa, 2023.

Breast and cervical cancers show the highest incidence in East-Central Africa (>80 per 100,000), with Equatorial Guinea, Gabon, and Malawi as epicentres. Cervical cancer demonstrates pronounced Eastern African dominance, particularly in Eswatini and Malawi. Ovarian and uterine cancers remain geographically diffuse, with a lower burden (<15 per 100,000), exhibiting modest regional variation. Breast cancer represents the predominant female malignancy across sub-Saharan Africa, followed by cervical cancer, highlighting regional priority differences for cancer control interventions and screening resource allocation, as shown in Figure 5.

**Figure 6:**
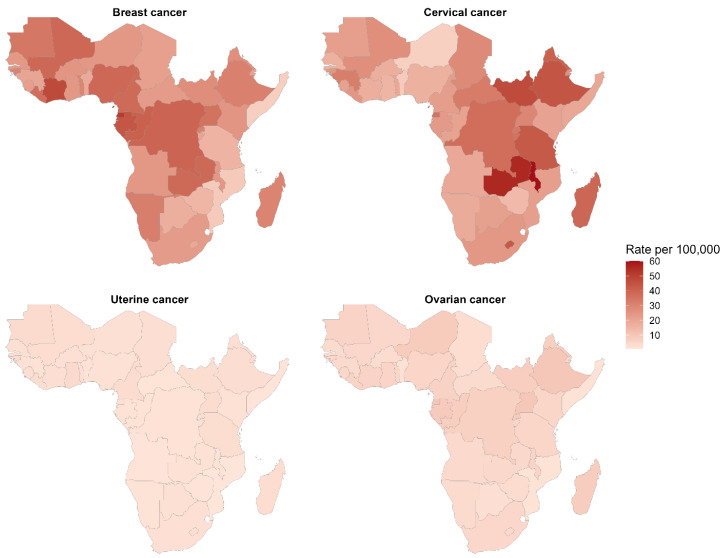
Age-standardised mortality rates (deaths per 100,000) for breast, cervical, uterine, and ovarian cancers in sub-Saharan Africa, 2023.

As shown in Figure 6, breast and cervical cancer mortality demonstrates the most pronounced geographic concentration in Central and East Africa, with death rates exceeding 35–50 per 100,000 in hyper-endemic zones, including the DR Congo, Uganda, Zambia, Malawi, and Eswatini. Cervical cancer mortality shows sharper spatial clustering, with darker shading concentrated in a band across East-Central Africa (Malawi, Zambia, and Tanzania), indicating concentrated treatment access gaps and advanced-stage disease predominance. Uterine and ovarian cancer mortality remains substantially lower and more geographically diffuse across all regions (<10 per 100,000).

**Figure 7:**
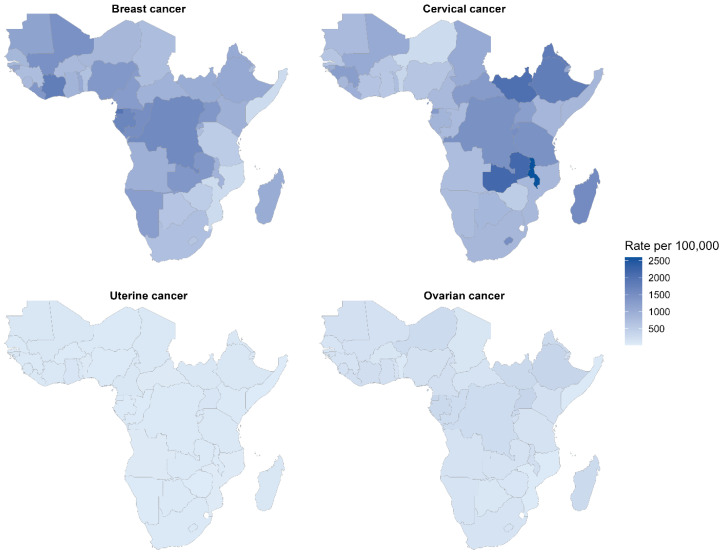
Age-standardised disability-adjusted life years rates for breast, cervical, uterine, and ovarian cancers in sub-Saharan Africa, 2023.

As shown in Figure 7, breast cancer demonstrates the highest DALYs burden across SSA (1000–2500 per 100,000), with concentrated dark blue shading in Central and East Africa indicating catastrophic disease impact in Equatorial Guinea, the Democratic Republic of Congo, Gabon, Uganda, and Malawi. Cervical cancer exhibits the second-highest DALYs concentration with pronounced East-Central African clustering (1000–2000 per 100,000), particularly in Eswatini, Malawi, and Zambia, reflecting combined incidence and case fatality burden. Uterine and ovarian cancers exhibit markedly lower DALYs burden (<500–1000 per 100,000), with minimal geographic variation and predominantly pale blue colouration across all regions. The DALYs distribution integrates both premature mortality and morbidity burden, revealing that breast and cervical cancers disproportionately drive overall female cancer disease burden across sub-Saharan Africa, with hyper-endemic zones experiencing > 2000 DALYs per 100,000 population.

**Age distribution of incidence, mortality, and disability-adjusted life years for female cancers in sub-Saharan Africa (2023)**

**Figure 8:**
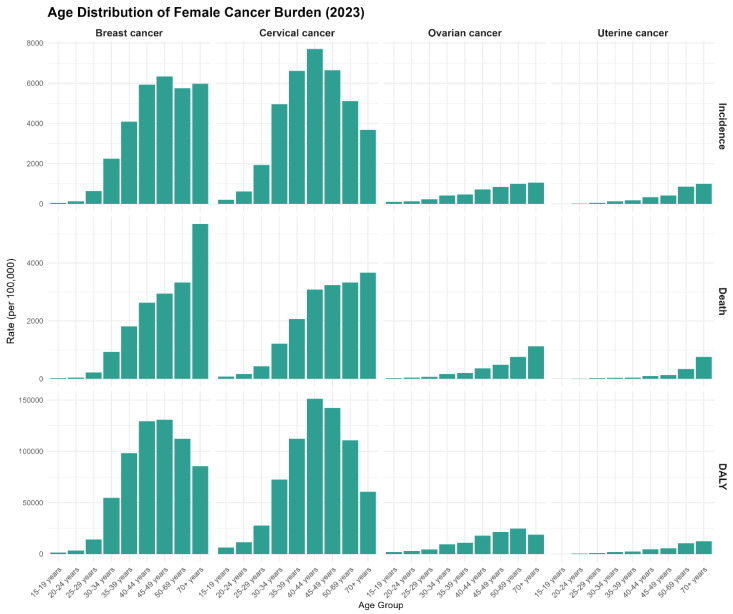
Age distribution of incidence, mortality, and disability burden for breast, cervical, uterine, and ovarian cancers in sub-Saharan Africa, 2023.

Breast cancer exhibits a broad age distribution, with peak incidence at 50–64 years (approximately 6000 per 100,000), declining sharply after age 70, demonstrating a typical post-menopausal pattern reflecting hormonal risk factors and ageing-related pathogenesis. Mortality follows incidence patterns with peaks in 50–64 age groups (approximately 3500 per 100,000), while DALYs remain elevated across 40–70 years, indicating substantial disease burden throughout mid-to-late life. Cervical cancer demonstrates an earlier peak onset at 40–49 years (approximately 7500 incidences per 100,000), with a sharper post-60 decline indicating reduced sexual activity and screening access gaps in older women. Mortality peaks similarly at 40–49 years (approximately 3500 per 100,000), while DALYs remain elevated through 60+ years. Ovarian cancer shows much lower absolute incidence and mortality (peaks < 800 per 100,000) with flatter age distribution, suggesting less age-dependent risk dynamics. Uterine cancer exhibits the youngest age-of-onset distribution, with substantial burden beginning at 35–39 years. These age-stratified patterns highlight that cervical cancer predominantly affects reproductive-age women (40–49 years), breast cancer affects post-menopausal women (50–70 years), and uterine cancer shows an intermediate but broader age distribution, informing age-targeted screening and intervention strategies across SSA, as Figure 8 indicates.

### Risk Factors

Table 5 indicates that risk factor attribution reveals divergent patterns across cancer types and regions. Cervical cancer demonstrates overwhelming dominance of unsafe sex as a risk factor, accounting for 1040.90 DALYs per 100,000 regionally, with Eastern Africa experiencing the highest burden at 1470.57 DALYs, underscoring the persistent HPV-related disease burden despite preventable aetiology. The breast cancer burden is distributed across multiple modifiable risk factors, with dietary risks contributing 106.97 DALYs per 100,000 (the highest among all risk factors), followed by tobacco (47.48 DALYs) and high fasting plasma glucose (47.77 DALYs). Regional disparities show Central sub-Saharan Africa experiencing elevated breast cancer risk from high fasting plasma glucose (104.93 DALYs) and tobacco (69.17 DALYs), suggesting metabolic and smoking-related disease predominance, while Southern Africa shows lower dietary risk (92.61 vs 126.12 DALYs in Western Africa). Uterine cancer burden attributable to high body mass index (19.03 DALYs regionally) remains substantially lower than breast cancer but shows Southern African elevation (25.13 DALYs), reflecting obesity-driven pathophysiology concentrated in wealthier sub-regions. Ovarian cancer demonstrates minimal attributable burden from measurable risk factors (13.92 DALYs from BMI), suggesting that the current Global Burden of Disease methodology captures limited modifiable exposures for this cancer type. There is a higher dietary risk attribution for breast cancer in Western Africa (126.12 DALYs per 100,000) compared with Southern Africa (92.61 DALYs per 100,000). There is also a negligible occupational risk contribution to ovarian cancer (0.44 DALYs per 100,000 regionally, and 0 in Western Africa).

More results are included in the Appendix A.

## 4. Discussion

In this study, we utilised data from the 2023 GBD study to provide a retrospective ecological analysis of the breast, cervical, uterine, and ovarian cancer burden. Overall, the study confirms a general and significant escalation in incidence and mortality rates for the selected cancers. Breast cancer represents the highest and most rapidly accelerating cancer burden in SSA. Between 1990 and 2023, the overall regional incidence of breast cancer increased by 122% and mortality by 83%. This surge is most pronounced in Central SSA, which recorded the steepest acceleration in incidence (186%) and mortality (135%), culminating in the highest regional burden in 2023. The burden of breast cancer was distributed across multiple modifiable risk factors, with dietary risks contributing the highest burden (106.97 DALYs per 100,000), followed by high fasting plasma glucose (47.77 DALYs) and tobacco (47.48 DALYs). This finding is consistent with the study by Li et al. [21], which found that globally, dietary risk, high BMI and high fasting plasma glucose were significant risk factors that drive the burden of breast cancer. It highlights the need for SSA countries to invest in health interventions that promote healthy weight management, a balanced diet, and the regulation of fasting plasma glucose levels. Practically, this may include promoting physical activity, improving sleep, reducing salt intake, and limiting the consumption of ultra-processed foods [22,23,24].

For cervical cancer, the highest burden was reported in Eastern SSA. Despite being largely preventable, the persistently high case fatality ratios across the region, especially in countries like Eswatini (44.0%), Malawi (44.2%), and South Sudan (47.4%), underscore critical gaps in the cancer care continuum. This suggests that many women are still diagnosed at late stages, where treatment is less effective. Our analysis reveals a significant heterogeneity in trends, offering a model for success. A deeper interrogation of the findings suggests that cervical cancer DALYs are driven by unsafe sex, which accounts for 1040.90 DALYs per 100,000 population and thus underscores the persistent dominance of HPV-related pathology in the region. Nonetheless, countries like Mozambique and Tanzania have demonstrated a significant decline in both incidence and mortality, likely reflecting the positive impact of national HPV vaccination programmes and improved screening infrastructure [17]. Although unsafe sex dominates the risk profile, tobacco use adds a modest but significant contribution, accounting for 33.81 DALYs per 100,000 regionally and reaching its highest levels in Southern and Eastern Africa. These findings corroborate studies by Li et al. [18], Cheng et al. [25], Qui et al. [26], and Teng et al. [27], which found unsafe sex and tobacco use to be significant attributable risk factors for cervical cancer.

The study further reveals that uterine and ovarian cancers show a more modest, yet significant, escalation in burden, with uterine cancer incidence rising by 72% and mortality by 38% across the region. While their DALY burden is lower than that of breast and cervical cancers, the upward trajectory signals a growing future challenge that requires proactive, primary prevention strategies focused on lifestyle modification. It was observed that high BMI was the primary factor accounting for uterine and ovarian cancer DALYs. For ovarian cancer, high BMI accounted for 13.92 DALYs per 100,000 population; in the case of uterine cancer, high BMI accounted for 19.03 DALYs per 100,000 population. Similar patterns have been reported at the global level, where high BMI was the major attributable risk factor for uterine and ovarian cancers [28,29]. However, it is important to acknowledge that evidence supporting BMI-reduction interventions in many SSA settings remains limited [30]. As such, context-specific and age-sensitive strategies are warranted, particularly given that breast cancer in the region frequently presents at younger ages. Strengthening early detection approaches tailored to younger populations may therefore be as critical as addressing metabolic risk factors.

The risk factors show that the attribution patterns are broadly aligned with global GBD estimates, where unsafe sex remains the dominant driver of cervical cancer, and metabolic risks increasingly contribute to breast and uterine cancers. The higher dietary risk attribution observed in Western compared with Southern Africa likely reflects regional variation in nutrition transition, urban dietary shifts, and exposure prevalence rather than biological differences [30]. The negligible occupational risk contribution to ovarian cancer is consistent with global findings and may reflect limited quantifiable occupational carcinogen exposure within the GBD framework rather than a true absence of occupational determinants. Additionally, the geographic distribution for these cancers was less concentrated, suggesting that the underlying risk factors are more broadly distributed across the sub-regions.

### 4.1. Strengths and Limitations

The primary strength of this study lies in its use of the comprehensive GBD 2023 database, which provides standardised, long-term (1990–2023) estimates of incidence, mortality, and DALYs across all 48 SSA countries. This robust methodology enables valid temporal and cross-regional comparisons, providing an unprecedented level of detail on the evolving cancer landscape. The inclusion of a risk factor attribution analysis further strengthens the study by identifying key modifiable targets for prevention. Nevertheless, several limitations must be acknowledged. First, as an ecological study based on GBD estimates, the findings are subject to the inherent limitations of the GBD methodology, including reliance on statistical modelling to address data sparsity in some SSA countries. This may introduce uncertainty, as reflected in the 95% UIs. These UIs are presented to describe estimation uncertainty in modelled outputs; no formal statistical inference was conducted and overlap or non-overlap of UIs should not be interpreted as evidence of statistical significance. Also, the ecological design precludes the establishment of individual-level causal relationships. The GBD data do not include information on cancer stage at diagnosis, which is a critical determinant of mortality and would have provided a more complete picture of the quality of cancer care.

### 4.2. Implications for Policy and Research

Essentially, the findings suggest that a one-size-fits-all approach may not be the most effective way to combat breast, cervical, uterine, and ovarian cancers in SSA. Each SSA country must develop unique interventions for the selected cancers. Countries exhibiting declining or stabilising trends, particularly for cervical cancer, provide empirical evidence that sustained investments in HPV vaccination, screening, and early treatment can yield measurable population-level gains. Conversely, the persistently high mortality-to-incidence ratios across most settings point to late-stage presentation and limited access to definitive treatment, which must be addressed through decentralised oncology services, workforce training, and referral system strengthening. In the case of cervical cancer, the persistent dominance of HPV-related disease and alarmingly high case fatality ratios highlight the need for a comprehensive scale-up of HPV vaccination, expansion of organised screening programmes, and investment in treatment capacity, including radiotherapy and pathology services. Additionally, promoting education and advocating for safe sex practices could prove effective in reducing the incidence of cervical cancer. For breast cancer, policies should strengthen population-level interventions addressing dietary patterns, obesity prevention, and glycaemic control, alongside sustained tobacco control efforts.

### 4.3. Conclusions and Future Research Recommendations

Between 1990 and 2023, SSA has experienced a consistent increasing trend in the incidence, mortality and DALYs for breast, cervical, uterine and ovarian cancers. We conclude that while the burden of breast cancer is highest in Central SSA, cervical cancer is pervasive in Eastern Africa. Without targeted interventions for the different cancers, SSA countries will continue to experience increased incidence and mortality. As such, we call for the identification of the right risk factors to be targeted for each cancer. Priority should be on promoting healthy weight management, healthy dieting and regulation of fasting plasma glucose as an approach to reduce the breast cancer incidence. For cervical cancer, the promotion of HPV vaccination and safe sex practices could be instrumental in positioning SSA toward a declining trend in cervical cancer incidence. While highlighting these preventive measures, SSA countries must commit resources to secondary prevention models, such as integrating cancer screening into primary healthcare service provision. This will promote early detection, contribute to reducing mortality, and improve DALYs. Future research should consider country-specific and subnational analyses that move beyond ecological trends to examine health system determinants of cancer outcomes, including diagnostic delays, treatment pathways, and financial barriers to care. Additionally, future studies should assess the effectiveness, scalability, and equity impacts of existing screening and vaccination programmes, with a particular focus on rural populations and marginalised groups.

## Figures and Tables

**Table 1:** Breast cancer incidence rates, death rates, and disability-adjusted life years in sub-Saharan Africa by region and country, 1990 to 2023.

Location	Incidence Rate 2023 (95% UI)	Incidence % Change 1990–2023	Death Rate 2023 (95% UI)	Death % Change 1990–2023	DALYs Rate 2023 (95% UI)	DALYs % Change 1990–2023
**Sub-Saharan Africa**	53.57 (38.90–71.11)	122.1703	29.68 (21.16–38.99)	83.03204	1055.52 (753.79–1375.54)	86.31357
**Eastern**	44.36 (30.80–58.19)	120.9921	25.03 (17.25–33.35)	83.49648	889.00 (612.00–1171.52)	80.01682
Ethiopia	54.82 (37.00–79.54)	301.8095	31.32 (21.45–42.25)	206.9721	1062.62 (703.68–1535.36)	201.6039
Somalia	11.15 (6.67–16.89)	79.81868	7.55 (4.62–11.38)	65.04774	269.77 (163.67–403.85)	70.12585
Burundi	33.42 (21.38–46.84)	78.28561	20.08 (12.68–28.19)	56.58208	744.92 (468.86–1027.24)	49.64566
Djibouti	36.87 (24.20–52.43)	39.95834	20.62 (13.35–28.40)	21.63777	747.97 (485.67–1059.84)	17.61609
Kenya	49.73 (35.58–65.63)	105.6907	25.17 (17.50–33.38)	71.66799	943.30 (655.14–1234.09)	76.76167
Madagascar	48.55 (32.96–66.45)	36.73461	30.23 (20.19–40.94)	25.18946	1015.25 (675.55–1386.41)	14.58813
Malawi	41.76 (27.24–60.64)	94.65771	24.21 (15.57–34.88)	72.87594	904.63 (587.44–1309.10)	63.5045
Mozambique	12.83 (8.72–18.17)	−0.4134	8.99 (6.01–12.69)	0.486471	265.82 (178.39–382.47)	−17.8878
Rwanda	54.33 (37.11–75.92)	76.51362	31.21 (21.55–43.47)	50.52235	1084.98 (741.33–1533.33)	27.51862
South Sudan	43.51 (28.18–60.81)	69.1842	27.13 (17.70–37.42)	56.60119	1015.49 (653.50–1404.35)	56.82467
Uganda	68.73 (46.29–90.92)	203.0617	35.45 (23.96–46.10)	135.2565	1364.32 (954.77–1787.28)	157.8477
Zambia	65.75 (45.11–92.82)	120.7782	38.41 (25.88–53.81)	88.14799	1355.04 (925.72–1906.01)	85.51705
Eritrea	45.68 (30.89–62.63)	142.2864	27.00 (18.46–36.83)	102.0735	1051.21 (700.80–1444.89)	108.8413
Comoros	14.09 (9.52–19.45)	53.38519	8.63 (5.76–11.75)	34.6389	277.68 (189.37–379.64)	25.39479
Tanzania	26.15 (16.97–36.54)	17.58841	15.94 (10.33–22.46)	5.889513	503.05 (318.20–709.14)	−3.53107
**Western**	61.83 (44.37–86.19)	114.4527	34.02 (24.36–46.88)	76.33422	1197.12 (863.76–1640.01)	77.62087
Burkina Faso	39.63 (25.42–54.08)	78.9332	24.96 (16.35–33.52)	51.47735	798.59 (533.93–1069.03)	60.79683
Nigeria	72.11 (50.63–103.81)	108.7778	38.73 (26.06–56.34)	73.46339	1341.22 (926.22–1935.41)	66.77077
Mauritania	66.17 (41.97–96.50)	228.9062	34.26 (22.76–49.02)	140.9876	1164.27 (742.17–1686.20)	154.5901
Benin	33.33 (22.80–47.61)	103.1956	18.50 (12.96–26.57)	58.5977	674.03 (464.74–964.87)	74.82986
Cabo Verde	41.80 (27.96–58.51)	111.7766	18.99 (13.16–25.95)	48.03625	612.99 (423.97–844.48)	58.87831
Côte d’Ivoire	86.65 (58.62–116.53)	153.7763	47.29 (31.32–63.63)	97.72851	1776.78 (1193.79–2408.52)	127.3456
Ghana	45.28 (33.13–59.99)	64.59639	24.14 (18.03–31.72)	34.91993	881.39 (653.85–1152.07)	35.53243
Guinea	33.84 (23.05–49.37)	106.5374	20.25 (13.77–28.91)	69.76546	738.82 (499.86–1068.04)	89.07417
Guinea-Bissau	47.31 (32.30–66.80)	154.3688	30.48 (21.35–42.63)	119.0821	1087.65 (748.35–1540.87)	128.3951
Liberia	67.08 (46.62–94.55)	192.7791	37.01 (25.23–52.09)	129.3246	1353.21 (925.66–1887.63)	149.5716
Mali	67.96 (43.70–95.62)	187.1847	38.74 (25.02–53.62)	132.8521	1464.39 (925.11–2057.28)	152.6504
Senegal	41.98 (28.20–58.76)	161.8703	23.77 (15.84–33.29)	117.2761	826.43 (549.52–1153.38)	114.831
Sierra Leone	38.68 (25.97–52.83)	88.61915	22.09 (14.81–30.27)	59.0405	805.39 (537.31–1113.96)	63.70304
Gambia	34.82 (22.90–50.47)	128.843	20.50 (13.74–29.71)	98.46019	701.74 (460.01–1012.70)	99.56751
Niger	39.74 (26.72–58.64)	69.27973	24.10 (16.00–34.99)	43.31356	838.16 (559.11–1247.74)	49.51841
Togo	51.34 (33.98–70.96)	120.5197	29.72 (20.53–40.16)	81.42712	1047.39 (708.67–1451.56)	95.56267
**Central**	64.85 (41.77–91.00)	186.0497	35.56 (23.13–50.25)	135.4856	1395.89 (910.58–1971.31)	142.7628
Central African Republic	36.72 (20.76–56.49)	68.73633	23.34 (13.33–35.41)	52.85631	916.19 (523.22–1404.87)	57.39765
São Tomé and Principe	49.11 (31.49–69.11)	196.5263	25.09 (15.82–35.82)	126.7324	857.38 (536.48–1199.99)	139.3433
DR Congo	73.02 (44.02–105.12)	215.8763	39.90 (24.36–57.64)	160.0464	1584.71 (968.21–2295.20)	172.1979
Gabon	88.82 (60.43–125.08)	159.806	43.88 (30.61–60.21)	105.3459	1660.64 (1156.91–2279.01)	103.0693
Angola	44.09 (28.47–64.15)	151.4756	24.40 (15.98–34.22)	104.1741	946.18 (610.14–1364.58)	107.952
Cameroon	65.13 (44.74–89.07)	85.92794	37.96 (25.82–52.16)	55.20762	1268.18 (861.90–1726.52)	55.76551
Chad	33.01 (20.57–48.08)	51.70989	21.64 (13.61–31.22)	37.43441	731.66 (458.23–1062.55)	44.71638
Equatorial Guinea	107.01 (67.94–151.05)	354.2266	51.26 (32.21–71.96)	211.9673	1951.06 (1218.84–2712.93)	213.7439
Congo	73.73 (49.84–110.70)	135.2368	40.69 (27.67–61.23)	102.295	1502.19 (979.04–2299.67)	86.34587
**Southern**	39.59 (32.51–47.67)	84.20433	22.08 (18.26–25.90)	56.46792	690.85 (553.99–834.83)	62.29067
Eswatini	37.68 (23.46–59.89)	−5.6169	20.35 (13.12–31.49)	−12.56	836.63 (508.53–1325.49)	−16.9285
Lesotho	28.47 (18.09–41.31)	24.50763	19.06 (12.53–26.57)	20.10601	599.67 (385.88–878.33)	21.02652
Botswana	34.66 (21.70–52.33)	125.659	17.15 (11.10–25.18)	72.70619	626.98 (381.50–975.91)	78.63601
Namibia	62.97 (43.50–81.85)	96.72283	31.97 (22.64–40.92)	64.6234	1244.44 (825.66–1624.71)	58.07896
South Africa	41.88 (34.27–51.73)	84.3633	23.17 (19.23–27.19)	55.54402	706.56 (575.71–891.79)	61.07123
Zimbabwe	23.24 (15.49–32.99)	87.35187	14.93 (9.95–21.22)	73.33928	481.41 (314.52–694.46)	98.09427

**Table 2:** Cervical cancer incidence rates, death rates, and disability-adjusted life years in sub-Saharan Africa by region and country, 1990 to 2023.

Location	Incidence Rate 2023 (95% UI)	Incidence % Change 1990–2023	Death Rate 2023 (95% UI)	Death % Change 1990–2023	DALYs Rate 2023 (95% UI)	DALYs % Change 1990–2023
**Sub-Saharan Africa**	55.90 (39.69–78.01)	30.26433	27.11 (18.78–37.79)	14.9801	1040.90 (738.02–1452.50)	13.11352
**Eastern**	78.65 (54.87–108.76)	28.03219	37.03 (25.67–50.66)	12.96629	1470.57 (1042.60–2049.41)	10.04375
Ethiopia	97.93 (54.16–166.79)	96.08568	44.16 (25.84–73.59)	62.20307	1782.25 (1013.95–2909.85)	55.03489
Somalia	38.25 (20.97–65.37)	51.20408	19.58 (11.34–32.91)	43.99675	827.90 (483.13–1390.48)	44.59136
Burundi	77.03 (46.25–121.64)	55.92646	35.50 (21.20–53.96)	42.40994	1523.25 (906.75–2311.23)	35.20116
Djibouti	60.28 (33.65–105.06)	12.03497	26.22 (15.58–44.32)	1.482674	1100.08 (634.44–1886.82)	−0.92607
Kenya	48.02 (30.87–70.09)	4.057078	20.94 (13.63–30.66)	−8.77922	847.38 (545.56–1216.32)	−8.32412
Madagascar	80.22 (49.32–130.73)	−2.25307	39.03 (23.87–61.74)	−6.64471	1557.62 (949.47–2491.77)	−12.1331
Malawi	135.87 (64.29–228.46)	80.7114	60.03 (30.04–98.56)	59.12059	2598.73 (1264.11–4312.86)	59.0115
Mozambique	41.12 (24.69–61.78)	−34.1669	21.94 (13.45–33.53)	−26.27	822.36 (487.00–1226.97)	−38.596
Rwanda	74.94 (37.81–128.72)	−0.39261	33.40 (17.25–56.10)	−8.18954	1367.43 (687.42–2251.86)	−19.5348
South Sudan	99.27 (57.94–163.64)	55.77962	47.05 (28.32–72.51)	48.20316	2026.36 (1217.58–3174.78)	47.43436
Uganda	69.81 (42.26–111.67)	47.0153	29.21 (18.59–46.05)	18.11896	1228.73 (776.90–1890.17)	24.22349
Zambia	112.59 (68.22–172.17)	40.27084	55.85 (35.66–85.78)	28.80666	2163.81 (1389.75–3327.66)	25.28267
Eritrea	93.60 (57.90–153.97)	69.16095	40.69 (25.58–62.85)	43.44953	1816.26 (1136.20–2846.68)	45.41817
Comoros	16.92 (9.46–30.97)	−20.5745	9.01 (5.20–15.30)	−23.9889	331.31 (189.21–565.00)	−29.2818
Tanzania	76.21 (50.69–111.50)	−18.2943	42.98 (27.86–62.41)	−22.1294	1448.28 (959.55–2096.61)	−27.12
**Western**	35.68 (22.89–52.81)	6.711382	17.82 (11.96–25.12)	−6.93989	660.44 (448.86–947.11)	−6.42364
Burkina Faso	30.57 (19.81–46.41)	−7.49436	17.47 (11.03–26.30)	−17.6559	588.54 (369.23–886.58)	−16.3003
Nigeria	33.88 (22.13–51.41)	−2.33864	16.61 (11.15–24.40)	−14.1495	610.60 (412.66–897.02)	−15.3491
Mauritania	46.44 (24.99–75.68)	92.29017	22.09 (12.63–34.09)	53.14341	793.66 (437.11–1264.83)	56.54585
Benin	18.93 (11.35–29.15)	29.98596	8.77 (5.68–13.04)	2.786837	343.35 (216.66–516.95)	9.45221
Cabo Verde	33.93 (18.93–57.75)	14.21036	15.57 (9.19–24.24)	−14.7113	512.69 (284.34–864.48)	−7.09264
Côte d’Ivoire	33.67 (20.43–52.10)	34.63922	17.22 (10.39–27.17)	11.2128	639.12 (394.84–999.33)	19.89989
Ghana	31.95 (16.46–58.76)	6.441176	15.34 (8.21–27.42)	−4.86363	578.51 (307.63–1032.72)	−7.34591
Guinea	62.28 (38.14–97.15)	17.16528	31.72 (20.06–49.38)	0.192679	1234.64 (770.96–1935.03)	5.628701
Guinea-Bissau	46.26 (27.70–78.48)	69.25746	24.54 (14.97–39.47)	52.01794	942.29 (571.78–1513.87)	52.61512
Liberia	51.82 (26.98–94.75)	66.13272	24.75 (12.94–44.02)	36.6518	958.13 (522.09–1692.14)	42.6042
Mali	53.96 (34.95–80.03)	23.51315	27.02 (17.65–39.81)	8.936609	1049.91 (692.82–1523.32)	8.964876
Senegal	33.55 (20.42–49.88)	32.79914	16.62 (10.47–25.27)	18.68057	616.93 (394.42–932.28)	14.45901
Sierra Leone	43.91 (23.62–72.50)	13.00269	21.47 (11.82–34.83)	0.578449	827.28 (464.48–1329.82)	0.625229
Gambia	25.80 (13.79–45.94)	36.83932	12.33 (7.17–21.65)	25.50372	475.96 (265.65–834.46)	23.89011
Niger	14.31 (8.74–24.32)	26.97046	7.18 (4.60–11.78)	10.79843	275.02 (168.53–464.97)	13.10172
Togo	38.57 (22.43–63.74)	24.76529	19.47 (11.62–32.18)	7.445646	729.35 (444.52–1218.79)	11.67595
**Central**	63.91 (37.63–101.57)	68.0862	31.37 (18.75–51.49)	46.82335	1228.85 (735.84–1939.54)	47.37569
Central African Republic	58.44 (28.52–96.58)	43.66394	32.77 (15.72–54.20)	33.67858	1285.10 (612.04–2137.27)	35.96198
São Tomé and Principe	52.19 (20.74–100.08)	22.98544	23.68 (9.50–45.05)	2.762506	887.07 (356.83–1698.75)	4.383345
DR Congo	76.33 (40.59–128.88)	88.3944	36.94 (20.46–65.62)	62.99919	1462.07 (830.80–2497.42)	65.64743
Gabon	49.09 (30.50–77.66)	62.61849	23.45 (15.68–36.57)	42.38717	878.44 (580.19–1379.23)	38.76999
Angola	38.27 (24.12–58.82)	32.42538	18.93 (12.52–27.44)	14.50098	733.10 (478.58–1072.67)	13.89898
Cameroon	42.99 (23.96–74.12)	9.433841	22.31 (13.01–37.07)	−3.78769	797.41 (463.66–1367.00)	−4.8955
Chad	51.49 (28.64–96.76)	12.31102	28.22 (15.59–51.24)	4.490154	1042.93 (585.40–1921.92)	6.737454
Equatorial Guinea	75.55 (42.22–117.25)	105.7381	35.58 (20.93–53.53)	64.38852	1329.24 (765.67–2009.81)	58.31772
Congo	40.02 (22.91–71.60)	10.77227	21.13 (12.61–37.27)	10.30324	765.60 (452.22–1365.92)	−1.54673
**Southern**	46.20 (33.39–65.71)	120.4571	23.75 (17.66–32.41)	97.14776	825.28 (608.58–1138.70)	100.5385
Eswatini	157.63 (88.71–250.28)	19.76011	69.44 (42.41–102.98)	20.0034	2999.15 (1759.74–4633.16)	12.87734
Lesotho	73.28 (33.88–128.43)	92.06	43.57 (20.02–75.40)	90.34297	1511.84 (685.23–2642.02)	91.82796
Botswana	51.01 (28.69–84.76)	109.8332	21.76 (13.19–32.40)	69.09776	840.51 (493.04–1339.88)	67.32055
Namibia	44.31 (24.92–69.64)	51.23965	18.35 (11.18–27.99)	32.92492	761.15 (455.80–1195.18)	29.08877
South Africa	47.54 (34.20–69.67)	153.1895	24.48 (18.15–34.05)	121.2158	834.46 (611.61–1199.66)	128.985
Zimbabwe	24.38 (14.87–37.62)	48.79447	13.95 (8.33–21.31)	37.57325	492.44 (298.11–754.80)	52.67094

**Table 3:** Ovarian cancer incidence rates, death rates, and disability-adjusted life years in sub-Saharan Africa by region and country, 1990 to 2023.

Location	Incidence Rate 2023 (95% UI)	Incidence % Change 1990–2023	Death Rate 2023 (95% UI)	Death % Change 1990–2023	DALYs Rate 2023 (95% UI)	DALYs % Change 1990–2023
**Sub-Saharan Africa**	8.82 (6.19–12.08)	108.9028	5.91 (4.20–8.09)	96.90186	201.42 (142.44–271.42)	94.54051
**Eastern**	10.52 (7.27–14.14)	114.1913	6.80 (4.67–9.32)	103.3018	242.33 (166.33–324.49)	96.89925
Ethiopia	15.63 (9.73–25.33)	301.0803	9.97 (6.24–16.17)	269.5227	358.25 (222.13–572.53)	254.2538
Somalia	2.67 (1.57–4.80)	52.27498	1.81 (1.02–3.27)	50.64789	67.27 (39.27–122.27)	50.03534
Burundi	8.34 (5.31–12.68)	48.84288	5.29 (3.33–8.15)	50.36579	201.57 (129.10–306.41)	38.15537
Djibouti	8.54 (5.80–12.53)	31.06965	5.34 (3.62–7.88)	27.68113	198.09 (135.02–289.48)	22.42555
Kenya	8.44 (5.52–11.95)	66.80555	5.28 (3.57–7.38)	58.4203	188.42 (124.58–266.04)	56.87603
Madagascar	11.71 (7.81–16.63)	25.83222	7.99 (5.38–11.44)	27.74079	277.24 (183.49–396.64)	17.50209
Malawi	9.50 (5.84–13.81)	109.0336	6.00 (3.68–8.85)	99.02206	227.53 (136.18–337.19)	94.70434
Mozambique	2.09 (1.30–2.96)	−9.06621	1.55 (1.02–2.19)	2.182802	48.77 (30.26–70.05)	−15.7666
Rwanda	13.39 (8.74–19.29)	55.65212	8.61 (5.68–12.55)	62.08684	308.57 (200.67–438.30)	35.60218
South Sudan	12.21 (7.86–18.24)	63.53081	7.81 (5.02–11.59)	60.77055	300.80 (194.41–440.79)	59.09458
Uganda	15.94 (10.33–23.25)	144.9489	9.83 (6.60–14.17)	119.4013	360.62 (234.75–529.76)	129.6267
Zambia	7.05 (4.70–9.76)	108.2866	4.73 (3.15–6.71)	102.5913	165.27 (108.91–227.86)	96.04373
Eritrea	11.84 (7.67–17.12)	125.0895	7.15 (4.74–10.38)	105.7305	286.25 (187.58–407.18)	108.7658
Comoros	3.53 (2.20–5.25)	38.7535	2.51 (1.56–3.70)	39.48476	83.11 (51.60–121.68)	29.29313
Tanzania	7.03 (4.88–9.52)	44.88557	5.22 (3.67–7.24)	43.83816	158.76 (112.05–216.17)	35.28565
**Western**	7.73 (5.27–10.66)	102.2774	5.36 (3.61–7.47)	92.10117	174.84 (116.71–242.91)	88.62666
Burkina Faso	4.51 (2.98–6.69)	88.6591	3.43 (2.27–5.06)	81.38142	101.91 (67.20–150.45)	81.29079
Nigeria	7.98 (5.38–11.13)	98.47746	5.55 (3.76–7.74)	92.44267	176.88 (117.96–247.84)	82.96623
Mauritania	8.56 (5.59–12.94)	219.5336	5.87 (3.88–8.85)	186.5015	187.44 (123.02–278.86)	191.255
Benin	3.16 (2.01–4.53)	89.08161	2.13 (1.39–3.09)	67.7532	71.95 (46.68–102.72)	78.32586
Cabo Verde	3.52 (1.43–5.46)	462.4679	2.32 (0.93–3.65)	399.7965	73.37 (29.58–114.50)	414.0573
Côte d’Ivoire	9.44 (6.30–13.04)	84.8943	6.27 (4.21–8.67)	64.55756	218.58 (145.74–302.46)	76.93388
Ghana	8.62 (5.13–12.89)	132.4361	5.83 (3.55–8.86)	122.1632	196.78 (118.87–297.92)	116.4926
Guinea	5.82 (3.82–8.53)	110.1039	4.03 (2.63–5.85)	89.04055	138.32 (90.83–199.81)	104.4309
Guinea-Bissau	6.77 (4.48–9.66)	167.3878	4.82 (3.25–6.91)	152.7299	163.21 (107.80–234.40)	155.3478
Liberia	9.11 (5.90–13.52)	191.4227	6.09 (3.96–9.07)	161.517	210.11 (133.96–313.01)	173.8829
Mali	7.33 (4.96–10.39)	57.74197	5.00 (3.42–7.08)	47.87729	172.05 (116.44–243.07)	47.60786
Senegal	5.60 (3.75–8.33)	176.0267	3.88 (2.58–5.71)	165.2588	127.37 (86.34–189.92)	155.3768
Sierra Leone	7.37 (4.95–10.90)	97.36881	5.00 (3.36–7.29)	86.51111	170.67 (114.60–250.62)	86.51226
Gambia	2.45 (1.48–3.71)	169.6845	1.70 (1.05–2.59)	154.8103	56.38 (34.63–85.10)	160.2246
Republic of the Niger	11.77 (7.87–16.81)	62.93703	8.38 (5.57–12.15)	52.11494	270.59 (180.10–387.41)	55.65083
Togolese Republic	7.02 (4.38–10.44)	132.0457	4.85 (3.06–7.19)	113.1335	162.38 (101.56–241.93)	121.9994
**Central**	9.50 (5.61–14.92)	148.2419	6.16 (3.61–9.62)	130.3621	223.49 (131.37–347.75)	132.8823
Central African Republic	5.38 (2.67–9.55)	53.06003	3.75 (1.89–6.64)	46.00371	136.57 (69.47–244.53)	49.29532
São Tomé and Principe	8.91 (4.64–13.96)	181.0438	6.16 (3.15–9.60)	157.1485	198.97 (103.98–306.70)	163.6585
DR Congo	10.60 (5.62–17.86)	164.9685	6.80 (3.59–11.47)	144.1519	249.91 (131.57–421.29)	150.2391
Gabon	13.23 (8.76–19.13)	141.8539	8.55 (5.77–12.29)	126.5417	297.57 (199.16–424.57)	121.0083
Angola	6.62 (4.18–9.66)	145.4567	4.32 (2.71–6.22)	125.8619	155.05 (95.79–224.40)	127.2736
Cameroon	9.24 (6.21–13.04)	102.7572	6.61 (4.42–9.46)	91.0928	209.86 (141.16–297.61)	88.45711
Chad	4.37 (2.60–7.38)	56.70179	3.21 (1.90–5.30)	52.80973	103.70 (61.48–170.94)	54.34065
Equatorial Guinea	15.54 (9.88–22.91)	301.3636	9.97 (6.34–14.41)	259.6855	348.25 (221.31–513.75)	250.4218
Congo	11.06 (7.16–16.59)	115.9127	7.58 (5.03–11.18)	120.7116	256.48 (164.24–380.19)	99.39521
**Southern**	6.37 (4.83–7.98)	77.15546	4.71 (3.65–5.84)	69.26495	139.08 (106.06–175.15)	68.66829
Eswatini	6.19 (4.01–9.19)	−20.455	3.71 (2.42–5.38)	−18.3252	147.59 (92.17–220.71)	−24.2648
Lesotho	5.90 (3.27–9.12)	22.65157	4.58 (2.54–6.95)	25.12742	138.53 (75.81–214.50)	22.38672
Botswana	4.38 (2.80–6.24)	77.47978	2.78 (1.85–3.79)	69.04494	95.28 (60.76–138.12)	61.60462
Namibia	6.25 (4.19–8.89)	39.08833	3.85 (2.60–5.43)	37.89897	141.51 (95.55–203.91)	27.76259
South Africa	6.72 (5.08–8.46)	85.08357	5.01 (3.94–6.14)	74.87643	144.79 (111.19–181.41)	74.79821
Zimbabwe	4.63 (3.13–6.70)	70.90123	3.48 (2.33–4.87)	62.89956	107.92 (71.82–152.21)	77.81056

**Table 4:** Uterine incidence rates, death rates, and disability-adjusted life years in sub-Saharan Africa by region and country, 1990 to 2023.

Location	Incidence Rate 2023 (95% UI)	Incidence % Change 1990–2023	Death Rate 2023 (95% UI)	Death % Change 1990–2023	DALYs Rate 2023 (95% UI)	DALYs % Change 1990–2023
**Sub-Saharan Africa**	5.30 (3.61–8.00)	72.11176	2.37 (1.67–3.56)	37.81502	67.67 (47.50–99.99)	39.03781
**Eastern**	5.77 (3.91–8.58)	80.57459	2.51 (1.74–3.77)	42.9976	75.87 (52.59–113.24)	42.71228
Ethiopia	7.30 (3.80–11.60)	244.1458	3.05 (1.59–5.03)	138.673	93.58 (50.12–150.69)	138.5803
Somalia	1.87 (1.12–3.21)	65.28867	1.02 (0.61–1.73)	46.99115	31.70 (18.72–55.16)	53.12594
Burundi	4.13 (2.18–7.09)	79.07398	1.89 (1.01–3.23)	49.27235	61.33 (32.67–103.49)	44.30756
Djibouti	5.68 (3.59–9.21)	27.09466	2.36 (1.51–3.82)	6.868465	74.75 (47.70–120.23)	4.406113
Kenya	4.84 (3.05–7.39)	40.1125	1.92 (1.27–2.86)	16.14794	58.88 (38.11–88.73)	18.13575
Madagascar	6.96 (4.59–11.29)	23.10764	3.29 (2.15–5.31)	8.827625	97.52 (64.89–155.97)	2.447841
Malawi	3.23 (2.04–5.05)	56.69949	1.40 (0.89–2.16)	32.67568	45.76 (29.14–71.65)	30.95552
Mozambique	1.47 (0.92–2.29)	−5.67994	0.80 (0.54–1.25)	−5.2157	21.63 (14.16–33.73)	−19.14
Rwanda	5.84 (3.49–9.57)	62.3889	2.46 (1.49–4.11)	29.21374	76.60 (47.73–124.87)	10.88715
South Sudan	6.04 (3.87–9.25)	32.23227	2.92 (1.97–4.44)	20.56731	95.48 (63.45–147.23)	21.67341
Uganda	8.82 (5.39–13.96)	98.14171	3.54 (2.24–5.55)	46.29505	110.31 (69.31–175.44)	58.12661
Zambia	4.54 (2.84–7.22)	80.89431	2.02 (1.36–3.15)	46.51783	61.17 (40.20–93.85)	46.15939
Eritrea	6.24 (4.02–9.62)	99.75492	2.82 (1.89–4.37)	55.45572	95.69 (63.63–146.87)	62.77724
Comoros	2.30 (1.49–3.49)	25.0612	1.02 (0.64–1.57)	4.205258	29.80 (19.49–45.65)	−0.83675
Tanzania	6.27 (4.11–9.71)	27.02533	3.01 (1.97–4.93)	10.11108	81.05 (54.55–122.52)	5.386665
**Western**	5.35 (3.52–8.22)	60.68656	2.47 (1.66–3.66)	29.10621	67.57 (45.56–99.76)	28.89034
Burkina Faso	4.25 (2.70–6.71)	35.86683	2.33 (1.48–3.57)	13.74936	57.63 (36.76–89.65)	16.76805
Nigeria	4.58 (2.85–7.35)	62.44539	2.02 (1.29–3.10)	29.22084	55.31 (35.89–84.89)	27.20436
Mauritania	9.04 (5.50–14.47)	149.8533	3.77 (2.39–5.80)	73.53378	100.82 (63.11–155.09)	78.91897
Benin	3.13 (1.92–4.80)	48.71079	1.43 (0.90–2.19)	11.46124	40.52 (25.44–60.93)	19.14698
Cabo Verde	7.82 (4.89–11.98)	73.96277	3.03 (1.93–4.76)	18.08191	71.95 (46.80–107.36)	24.56556
Côte d’Ivoire	6.01 (3.99–8.98)	54.37048	2.67 (1.75–3.81)	17.37159	78.37 (52.32–116.09)	27.86973
Ghana	9.22 (5.93–14.29)	71.38712	4.13 (2.67–6.59)	34.83447	112.75 (72.53–176.68)	36.72384
Guinea	4.10 (2.66–6.27)	57.99224	2.02 (1.36–3.05)	24.66655	58.42 (39.23–90.80)	35.57965
Guinea-Bissau	5.25 (3.28–8.18)	93.73031	2.93 (1.86–4.58)	61.08674	81.27 (51.34–127.04)	66.65571
Liberia	6.34 (3.82–9.95)	90.02998	2.86 (1.82–4.44)	42.71785	81.87 (50.76–128.62)	51.88077
Mali	6.68 (4.43–9.52)	51.07832	3.20 (2.13–4.52)	24.47096	92.41 (62.18–133.27)	25.16746
Senegal	5.26 (3.31–8.02)	107.0026	2.46 (1.63–3.81)	65.30705	66.96 (44.20–102.82)	64.21186
Sierra Leone	5.49 (3.55–8.26)	48.65656	2.65 (1.75–3.91)	22.42448	73.80 (48.55–111.05)	24.6516
Gambia	2.38 (1.41–3.74)	110.9237	1.13 (0.67–1.76)	73.79607	31.58 (18.49–50.68)	80.07149
Republic of the Niger	4.19 (2.71–6.23)	36.56256	2.17 (1.43–3.26)	12.23998	58.27 (38.00–86.68)	15.60024
Togolese Republic	5.79 (3.38–9.77)	56.84508	2.79 (1.65–4.66)	25.63968	76.95 (46.29–131.09)	32.08643
**Central**	3.77 (2.14–6.29)	115.046	1.66 (0.95–2.85)	68.62158	51.69 (30.05–88.51)	74.65535
Central African Republic	2.40 (1.33–4.36)	48.46629	1.28 (0.69–2.41)	31.01107	40.21 (22.13–75.65)	36.21684
São Tomé and Principe	10.30 (6.02–16.75)	89.99276	4.41 (2.62–7.40)	40.19052	113.47 (69.10–183.50)	45.61581
DR Congo	4.22 (2.29–7.38)	133.1122	1.84 (1.02–3.31)	82.30773	58.24 (32.52–103.00)	91.89255
Gabon	4.78 (2.96–7.68)	112.7803	1.83 (1.15–2.96)	61.83412	55.60 (35.11–89.71)	60.57693
Angola	2.66 (1.61–4.11)	83.77297	1.18 (0.75–1.89)	40.59841	36.42 (22.57–58.19)	44.225
Cameroon	7.06 (4.63–11.23)	39.50838	3.41 (2.27–5.25)	14.06754	90.42 (59.09–139.75)	13.29256
Chad	4.90 (2.96–7.62)	25.76331	2.79 (1.68–4.25)	13.36684	73.62 (43.89–111.83)	16.65504
Equatorial Guinea	6.03 (3.69–9.71)	231.6186	2.19 (1.37–3.51)	101.0874	67.19 (42.39–107.75)	104.6878
Congo	3.61 (2.29–5.60)	92.73597	1.60 (1.00–2.48)	58.97088	46.97 (29.74–74.01)	48.54216
**Southern**	4.95 (3.38–7.13)	66.21731	2.25 (1.52–3.32)	45.38968	56.89 (39.44–82.98)	44.00986
Eswatini	8.27 (5.46–13.23)	−18.0545	3.55 (2.31–5.54)	−21.5114	116.03 (76.12–190.36)	−27.3488
Lesotho	4.43 (2.67–6.95)	−0.55444	2.49 (1.54–3.78)	−1.88013	64.00 (38.27–99.64)	−2.41171
Botswana	5.47 (3.20–8.68)	75.52106	2.22 (1.27–3.67)	30.92377	61.78 (36.41–96.93)	31.42187
Namibia	4.20 (2.64–6.37)	21.3891	1.70 (1.09–2.47)	0.170507	51.49 (33.47–76.90)	−4.79018
South Africa	5.36 (3.64–7.68)	77.07195	2.42 (1.62–3.58)	54.50691	60.01 (41.41–87.44)	54.26919
Zimbabwe	2.17 (1.43–3.42)	28.97396	1.18 (0.79–1.82)	24.21226	30.59 (20.33–46.25)	33.58819

**Table 5:** Age-standardised disability-adjusted life year rates (per 100,000) in 2023 for breast, uterine, cervical, and ovarian cancers among females in sub-Saharan Africa, by risk factor and region.

Types	Risk Factor	Sub-Saharan Africa	Central	Eastern	Southern	Western
Breast cancer	High body mass index	15.2984485	4.69917645	3.43310928	32.6321844	22.2720064
Breast cancer	High fasting plasma glucose	47.7717889	104.933177	19.4237611	45.3094341	56.4560316
Breast cancer	High alcohol use	18.416044	30.9291575	17.4362147	21.269762	14.8635165
Breast cancer	Tobacco	47.4786865	69.1680148	40.37292	63.6424936	42.0191391
Breast cancer	Dietary risks	106.96542	89.6058532	93.3118962	92.60924	126.119691
Uterine cancer	High body mass index	19.0283704	14.1251144	15.3870903	25.1301498	21.2326727
Cervical cancer	Tobacco	33.8145856	20.371289	51.0212434	76.3949307	10.2400783
Cervical cancer	Unsafe sex	1040.90283	1228.85388	1470.56655	825.275676	660.442036
Ovarian cancer	High body mass index	13.9150565	14.0108939	10.0098165	20.0448628	15.1794232
Ovarian cancer	Occupational risks	0.43705453	0.00207294	0.00727376	2.97107475	0

## Data Availability

All data generated and analysed during this study are available upon request and can also be downloaded online via https://vizhub.healthdata.org/gbd-results/, accessed on 20 December 2025.

## Abbreviations

ASIR: Age-standardised incidence rate; ASMR: Age-standardised mortality rate; BMI: Body mass index; CODEm: Cause-of-death ensemble model; DALY: Disability-adjusted life year; DisMod-MR 2.1: Disease Modelling Meta-Regression, version 2.1; DR Congo: Democratic Republic of the Congo; GBD: Global Burden of Disease; HPV: Human papillomavirus; NSCC: National Strategy for Cancer Control; PAF: Population-attributable fraction; SSA: Sub-Saharan Africa; UI: Uncertainty interval; YLD: Years lived with disability; YLL: Years of life lost.
